# Hypoxia and hypoglycaemia in Ewing's sarcoma and osteosarcoma: regulation and phenotypic effects of Hypoxia-Inducible Factor

**DOI:** 10.1186/1471-2407-10-372

**Published:** 2010-07-16

**Authors:** Helen J Knowles, Karl-Ludwig Schaefer, Uta Dirksen, Nicholas A Athanasou

**Affiliations:** 1 Botnar Research Centre, University of Oxford, Nuffield Orthopaedic Centre, Oxford, OX3 7LD, UK; 2 Univ Dusseldorf, Institute of Pathology, University Medical Centre Duesseldorf, D-40225 Dusseldorf, Germany; 3 Paediatric Haematology & Oncology, University Hospital Muenster, 48149 Muenster, Germany; 4 Department of Pathology, Nuffield Department of Orthopaedic Surgery, University of Oxford, Nuffield Orthopaedic Centre, Oxford, OX3 7LD, UK

## Abstract

**Background:**

Hypoxia regulates gene expression via the transcription factor HIF (Hypoxia-Inducible Factor). Little is known regarding HIF expression and function in primary bone sarcomas. We describe HIF expression and phenotypic effects of hypoxia, hypoglycaemia and HIF in Ewing's sarcoma and osteosarcoma.

**Methods:**

HIF-1α and HIF-2α immunohistochemistry was performed on a Ewing's tumour tissue array. Ewing's sarcoma and osteosarcoma cell lines were assessed for HIF pathway induction by Western blot, luciferase assay and ELISA. Effects of hypoxia, hypoglycaemia and isoform-specific HIF siRNA were assessed on proliferation, apoptosis and migration.

**Results:**

17/56 Ewing's tumours were HIF-1α-positive, 15 HIF-2α-positive and 10 positive for HIF-1α and HIF-2α. Expression of HIF-1α and cleaved caspase 3 localised to necrotic areas. Hypoxia induced HIF-1α and HIF-2α in Ewing's and osteosarcoma cell lines while hypoglycaemia specifically induced HIF-2α in Ewing's. Downstream transcription was HIF-1α-dependent in Ewing's sarcoma, but regulated by both isoforms in osteosarcoma. In both cell types hypoglycaemia reduced cellular proliferation by ≥ 45%, hypoxia increased apoptosis and HIF siRNA modulated hypoxic proliferation and migration.

**Conclusions:**

Co-localisation of HIF-1α and necrosis in Ewing's sarcoma suggests a role for hypoxia and/or hypoglycaemia in *in vivo *induction of HIF. *In vitro *data implicates hypoxia as the primary HIF stimulus in both Ewing's and osteosarcoma, driving effects on proliferation and apoptosis. These results provide a foundation from which to advance understanding of HIF function in the pathobiology of primary bone sarcomas.

## Background

Hypoxia is a fundamental micro-environmental component of solid tumour tissue which is associated with resistance to therapy, poor survival and a malignant phenotype [1]. Hypoxia induces stabilisation of the Hypoxia-Inducible Factor transcription factors, HIF-1 and HIF-2, which direct responses central to survival and expansion of the malignant cell population.

HIF comprises a hypoxia-inducible alpha subunit and a constitutively expressed beta subunit. Regulation of the active transcription factor occurs via enzymatic control of the abundance and activity of HIF*α *subunits. Under normoxia HIFα is post-translationally hydroxylated by the prolyl hydroxylase domain enzymes, targeting it for proteasomal degradation [2,3]. These enzymes are absolutely dependent on O_2 _and limitation of activity under hypoxia allows stabilisation of HIFα. Binding of the active complex to the hypoxia-response element (HRE) of target genes results in activation of pathways regulating processes such as angiogenesis, apoptosis and metabolic adaptation [4].

HIF over-expression is an independent prognostic factor in many carcinomas [5], although limited data is available in primary bone sarcomas. Nuclear over-expression of HIF-1α has been reported in approximately 60% of clinical osteosarcomas where it correlates with disease grade, stage, recurrence and survival [6-8]. In Ewing's sarcoma the presence of tumour cell-lined blood lakes correlates with clinical outcome. Cells surrounding these lakes also express HIF-1α and the hypoxia marker pimonidazole [9]. Expression of both HIF-1α and HIF-2α has been reported in chondrosarcoma [10] and giant cell tumour of bone [11], HIF-1α expression correlating with reduced disease-free survival in chondrosarcoma. Levels of serum VEGF, a HIF target gene, are significantly higher in Ewing's patients than healthy controls [12] and are an independent prognostic factor for survival [13]. In osteosarcoma VEGF levels were higher in the tumour and serum of patients who subsequently relapsed, tumour VEGF being predictive of pulmonary metastasis and poor prognosis [14,15]. In Ewing's sarcoma the presence of necrotic, non-perfused and presumably hypoxic tumour areas correlates with survival and frequency of metastatic spread [16-18].

This data implies that hypoxia and/or HIF contribute substantially to the pathobiology of primary bone sarcomas. Hypoxia induces expression of HIF-1α and VEGF in the osteosarcoma cell lines Saos2, 143B, U2-OS and MG-63 [11,19-21], with MG-63 also expressing HIF-2α [11,22]. The Ewing's sarcoma cell lines A673, SK-ES-1, SK-N-MC and TC-71 also demonstrate hypoxic induction of HIF-1α and downstream genes [23-25]. Despite such evidence for hypoxic activation of the HIF transcriptional cascade in osteosarcoma and Ewing's sarcoma cells, little is known regarding the effect of either HIF-1α or HIF-2α on the hypoxic phenotype of these cells. We have therefore analysed characteristics of the induction of HIF-1α, HIF-2α and HIF target genes in a panel of osteosarcoma and Ewing's sarcoma cell lines and investigated effects of isoform-specific HIF siRNA on the hypoxic phenotype of these cells.

## Methods

### Reagents

Tissue culture reagents were from Lonza (Wokingham, UK), except FBS (Invitrogen, Paisley, UK). Unless otherwise stated, reagents were from Sigma (Poole, UK). This study was approved by the Oxford Clinical Research Ethics Committee (C01.071).

### Immunohistochemistry

A tissue array comprising 47 Ewing's sarcomas was constructed at the University of Dusseldorf. Additional sections were from the Nuffield Orthopaedic Centre. Formalin fixed sections were stained for HIF-1*α *(BD Biosciences, Oxford, UK), HIF-2*α *(EP190b, Abcam, Cambridge, UK), Glut-1 (Abcam) and cleaved caspase 3 (Cell Signalling Technology, Danvers, USA). Staining was visualized with the EnVision™ Peroxidase/DAB Rabbit/Mouse detection kit (Dako, Ely, UK). Image acquisition was performed using an Olympus BX40 microscope with 20× or 40× objective, Olympus DP70 camera and CellF. Tumours were considered positive for an antigen when ≥ 3 positive cells/field of view were observed at 20× magnification.

### Cell culture

Cell lines were obtained from the EuroBoNeT cell line biobank, comprising recently characterised bone tumour cell lines [26], and maintained in culture for < 30 passages. All cell lines were maintained in RPMI (except TC-71; IMDM) with 10% FBS, L-glutamine (2 mM), penicillin (50 IU/ml) and streptomycin sulphate (50 μg/ml) in a humidified atmosphere at 37°C (5% CO_2 _in air). Hypoxic exposures were performed in 0.1% O_2_, 5% CO_2_, balance N_2 _in a MiniGalaxy incubator (RS Biotech, Irvine, UK). Low glucose conditions were achieved using RPMI media without glucose, supplemented as for normal media, under normoxic conditions.

### Western blotting

Cells were homogenized in lysis buffer (6.2 M urea, 10% glycerol, 5 M DTT, 1% SDS, protease inhibitors). Whole cell extract was separated by 8% SDS-PAGE and transferred to PVDF membrane. Primary antibodies were against HIF-1*α*, HIF-2*α*, Glut-1, Ki-67 (MIB-1, Dako) and β-tubulin. Immuno-reactivity was visualised with HRP-linked goat serum and chemiluminescence. Densitometric quantification of Ki-67, normalised to β-tubulin, was performed on scanned blots using ImageJ.

### Luciferase assay

Cells at 60% confluence were transfected with a PGK-HRE-firefly luciferase reporter construct and the control pHRG-TK renilla luciferase plasmid (Promega, Southampton, UK) using Lipofectamine 2000 (Invitrogen). 24 h post-transfection cells were exposed to experimental conditions then lysed in Passive Lysis Buffer. Firefly and renilla luciferase were assayed using the Dual-Luciferase Reporter Assay System (Promega), with firefly luciferase values normalized to the renilla transfection control.

### ELISA

Secretion of VEGF and TGFα was measured using the Human VEGF DuoSet and Quantikine Human TGFα Immunoassay (R&D Systems, Abingdon, UK) respectively.

### Cell number, proliferation and apoptosis

Cellular apoptosis was assessed using the Apo-One Homogeneous Caspase-3/7 Assay (Promega), with apoptosis levels normalised to total cell number (CellTiter 96 Aqueous One Solution Cell Proliferation Assay, Promega). For quantification of mitotic index cells were fixed in formalin, stained for H&E and the number of mitotic figures was expressed as a percentage of total cell number in 4 random fields of view.

### siRNA transfection

Small interfering RNA (siRNA) sequences against HIF-1*α *(H1), HIF-2*α *(H2) and HIF-1*α *scrambled control (scr) were as described [27] and obtained pre-annealed from Eurogentec. Cells at 40% confluence were transfected with 50 nM siRNA duplex using Lipofectamine 2000. Duplexes were removed after 24 h and cells treated as required.

### Scratch migration assay

A scratch was made through cells at 90% confluence using a 20 ul pipette tip. Specific points of the scratch were photographed before and after exposure to experimental conditions. Wound width was measured in CellF and migration expressed as fraction wound closure.

### Statistical significance

Results are expressed as mean ± SD of at least three independent experiments. Statistical analysis comprised one-way ANOVA using Bonferroni's multiple comparison as a *post-hoc *test, with results considered significant at p < 0.05.

## Results

### HIF-1α and HIF-2α are expressed in Ewing's sarcoma

**Figure 1:**
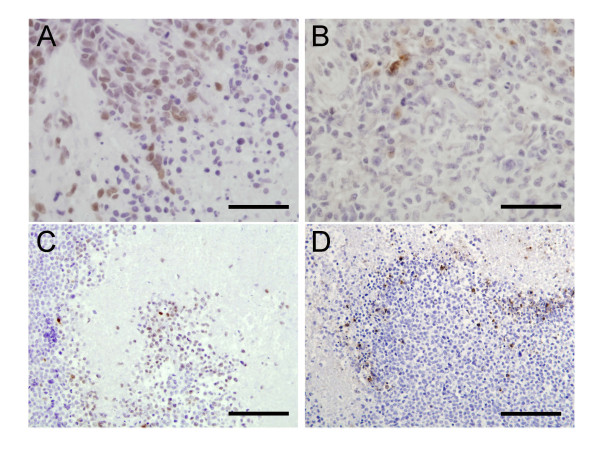
**Expression of HIF in Ewing's sarcoma**. (a) HIF-1α and (b) HIF-2α expression in Ewing's tumour cells; scale bar = 50 μm. Expression of HIF-1α (c) co-localises with that for cleaved caspase 3 (d); scale bar = 100 μm.

As HIF-1α has already been described to correlate with clinical and survival parameters in osteosarcoma, immunohistochemistry focussed on Ewing's sarcoma. Of 56 Ewing's tumours assessed for expression of HIF; 30% expressed only HIF-1α, 27% only HIF-2α, 18% both HIF-1α and HIF-2α and 25% neither HIF isoform. HIF-1α was predominantly localised in the nucleus of Ewing's tumour cells, whereas HIF-2α expression was mainly cytoplasmic (Figure 1A, B). In tumours expressing both HIF-1α and HIF-2α there was no evidence of co-localisation and no correlation in intensity of staining. In 17 of the 27 HIF-1α-positive cases expression of HIF-1α was associated with regions of necrosis, areas of tissue also immunoreactive for the apoptosis marker cleaved caspase 3 (Figure 1C, D). No such relationship was evident for HIF-2α. With the clinical data available, no correlation was observed between HIF expression and clinical parameters including tumour volume, metastasis and survival.

### Differential HIF response of ES and OS cells to hypoxia and low glucose

**Figure 2:**
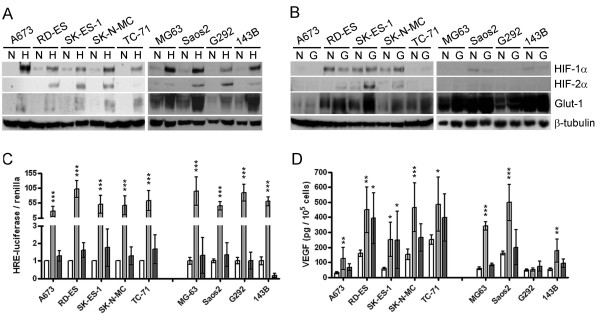
**HIF induction in ES and OS cell lines**. Western blots showing induction of HIF-1α (120 kDa), HIF-2α (120 kDa) and Glut-1 (50 kDa) in a panel of ES (A673, RD-ES, SK-ES-1, SK-N-MC, TC-71) and OS (MG-63, Saos2, G292, 143B) cell lines in response to 24 h (a) hypoxia (H; 0.1% O_2_) or (b) low glucose (G; over-exposed in comparison to hypoxia blots) compared with the untreated/normoxic (N) control. (c) Activation of the HRE-luciferase reporter construct and (d) secretion of VEGF in response to hypoxia (light grey bars) or low glucose (dark grey bars). *, p < 0.05; **, p < 0.01; ***, p < 0.001.

HIF-1α protein was stabilised in response to hypoxia in all Ewing's (ES) and osteosarcoma (OS) cell lines tested. Hypoxia induced the HIF-2α isoform in all OS and in 4/5 ES cell lines (Figure 2A). Low glucose conditions were also assessed as an additional micro-environmental characteristic of regions of necrosis. Low glucose produced no HIF response in any OS cell line, while ES cells displayed strong induction of HIF-2α protein and a moderate increase in HIF-1α in SK-ES-1 and SK-N-MC (Figure 2B). This pattern of HIF induction was reflected in transcriptional events downstream of HIF. Both ES and OS cells strongly transactivated the HRE-promoter element in response to hypoxia and induced expression of the HIF target genes, Glut-1 and VEGF (Figure 2A, C, D). ES cells induced expression of both Glut-1 and VEGF in response to low glucose, although the magnitude of induction was less than for hypoxia and no transcriptional activation was evident in the HRE promoter-luciferase assay (Figure 2B-D). OS cells showed no HIF transcriptional response to low glucose conditions.

### Distinct transcriptional effects of HIF-1α and HIF-2α in ES and OS cells

**Figure 3:**
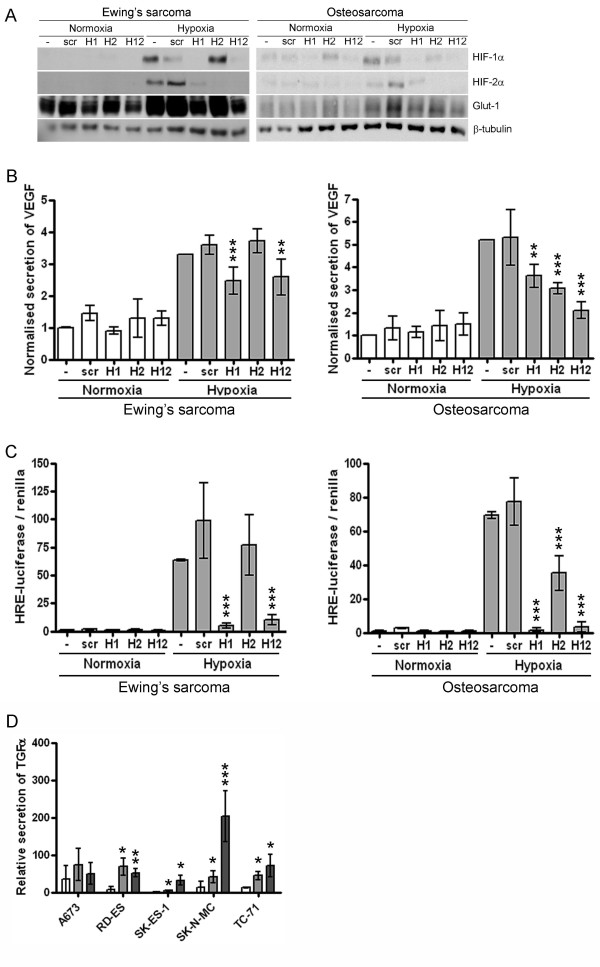
**HIF siRNA in ES and OS cell lines**. (a) Western blots showing expression of HIF-1α (120 kDa), HIF-2α (120 kDa) and Glut-1 (50 kDa) in response to siRNA targeting HIF-1α (H1), HIF-2α (H2), HIF-1α and HIF-2α (H12) or control siRNA (scr) in ES (SK-N-MC) and OS (143B) cells. Effects of HIF siRNA on (b) secretion of VEGF and (c) activation of the HRE-luciferase reporter construct and in response to hypoxia or low glucose in ES and OS cell lines. Bar graph data is normalised to the siRNA mock control (-) for each condition and represents pooled data from the entire panel of ES or OS cell lines. *, p < 0.05; **, p < 0.01; ***, p < 0.001 versus scr siRNA control. (d) Secretion of TGFα from ES cell lines in response to hypoxia (light grey bars) or low glucose (dark grey bars). *, p < 0.05; **, p < 0.01; ***, p < 0.001.

Isoform-specific HIF siRNA was used to distinguish the relative contribution of HIF-1α and HIF-2α to the HIF transcriptional response. In ES cell lines, HIF-2α siRNA had no effect on Glut-1 expression (Figure 3A), VEGF secretion (Figure 3B) or HRE-luciferase activity (Figure 3C). HIF-1α siRNA significantly inhibited hypoxic induction of Glut-1, VEGF and HRE transactivation (Figure 3A-C). Normoxic levels of Glut-1 and VEGF were also reduced by HIF-1α siRNA, even in the absence of immuno-detectable HIF (Figure 3A, B). However, hypoxia and low glucose both induced secretion of HIF-2-specific TGFα [28,29] in ES cell lines which express the HIF-2 isoform (Figure 3D). In contrast, HIF transactivation in OS cell lines was regulated by a combination of both transcription factors. Suppressed hypoxic induction of Glut-1 (Figure 3A), VEGF (Figure 3B) and HRE-luciferase activity (Figure 3C) was evident with siRNA targeting either HIF-1α or HIF-2α, combined 'HIF-1α plus HIF-2α' siRNA generally exhibiting an additive effect.

### Phenotypic effects of hypoxia and low glucose in ES and OS cell lines

**Figure 4:**
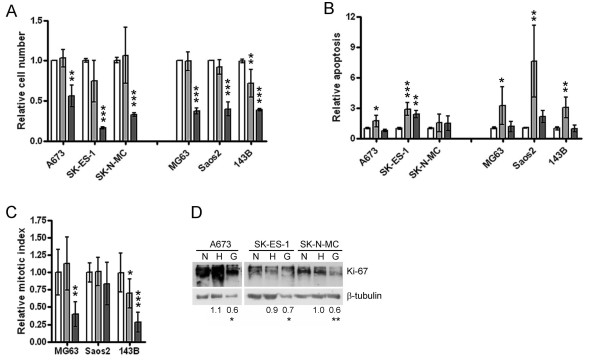
**Phenotypic effects of hypoxia and low glucose**. Effect of hypoxia (light grey bars) or low glucose (dark grey bars) on (a) cell number and (b) apoptosis in ES and OS cell lines and (c) mitotic index in OS cell lines. Bar graph data is normalised to the normoxic control (white bars) for each condition: *, p < 0.05; **, p < 0.01; ***, p < 0.001 versus control. (d) Proliferation assessed in ES cell lines by Western blot for Ki-67. N, normoxia; H, hypoxia; G, low glucose. Numerical values represent mean fold change in Ki-67 as assessed by densitometry:*, p < 0.05; **, p < 0.01 versus normoxia.

Phenotypic effects of 72 h exposure to hypoxia or low glucose conditions were assessed in a reduced panel of 3 ES and 3 OS cell lines. Hypoxia had no effect on total cell number in any cell line except 143B, whereas low glucose consistently reduced cell number by > 45% (Figure 4A). Caspase 3/7 activation was assessed as a marker of cellular apoptosis. Hypoxia increased apoptosis in both cell panels, having a 2 to 3-fold greater effect in OS cell lines. Glucose deprivation only had a significant effect on apoptosis in SK-ES-1 (Figure 4B). Proliferation was measured in OS cells by mitotic index and in ES cells by immuno-detection of Ki-67. The percentage of mitoses generally did not change during exposure to hypoxia, but was reduced by low glucose conditions (Figure 4C). Similarly, Ki-67 expression was inhibited by low glucose conditions but maintained on exposure to hypoxia (Figure 4D).

### Effect of HIF siRNA on the hypoxic phenotype

**Figure 5:**
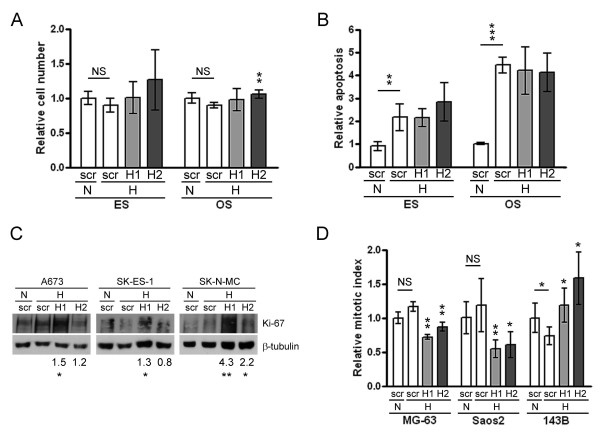
**Phenotypic effects of HIF siRNA**. Effect of HIF siRNA on (a) cell number, (b) apoptosis and (c, d) proliferation under hypoxia in ES and OS cell lines. Bar graph data is normalised to the relevant scrambled siRNA control (white bars): *, p < 0.05; **, p < 0.01; ***, p < 0.001; NS, not significant. (c) Proliferation assessed in ES cell lines by Western blot for Ki-67. N, normoxia; H, hypoxia. Numerical values represent mean fold change in Ki-67 as assessed by densitometry:*, p < 0.05; **, p < 0.01 versus hypoxic scrambled (scr) control.

Since no differences were observed between ES and OS cell lines regarding phenotypic effects of glucose deprivation, and as OS cells have no HIF transcriptional response to this stimulus, effects of HIF siRNA were only investigated under hypoxia. No effect was observed on cell number or apoptosis under hypoxia with the exception of a minor increase in OS cell number with HIF-2α siRNA, the magnitude of which questions its biological significance (Figure 5A, B). However, in ES cells HIF-1α siRNA consistently increased cell proliferation under hypoxia as assessed by Western blotting for Ki-67 (Figure 5C). Conversely, siRNA targeting either HIF-1α or HIF-2α resulted in reduced hypoxic proliferation in 2/3 OS cell lines, although siRNA targeting either HIF-1α or HIF-2α in 143B cells produced the opposite effect (Figure 5D).

### Effect of hypoxia and HIF on cell migration

**Figure 6:**
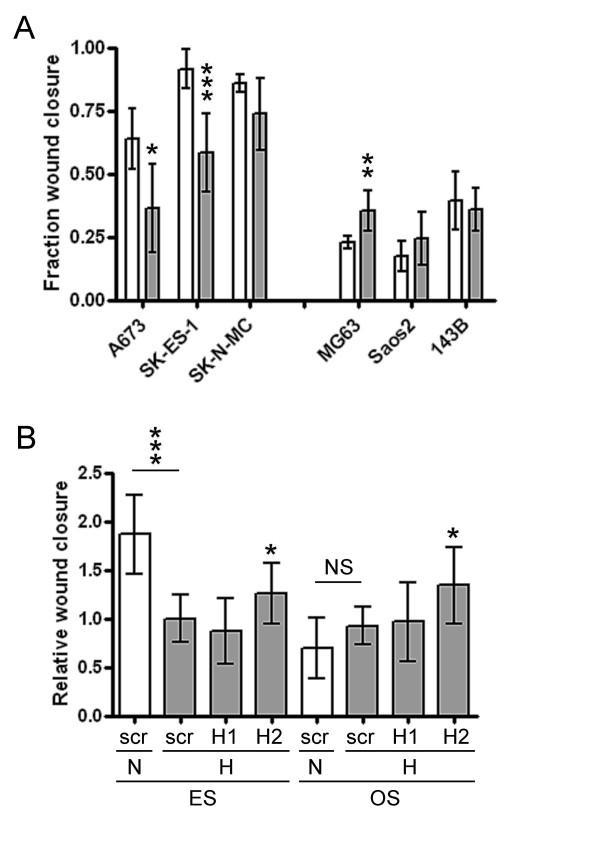
**Effect of hypoxia on migration of ES and OS cell lines**. (a) Fraction closure of a scratch wound due to cell migration during 18 h normoxia (white bars) or hypoxia (grey bars). (b) Effect of siRNA targeting HIF-1α (H1) or HIF-2α (H2) on migration of ES (combined data from A673, SK-ES-1 and SK-N-MC) or OS (combined data from MG63, Saos2 and 143B) cell lines under hypoxia. N, normoxia; H, hypoxia. Bar graph data is normalised to the (a) normoxic or (b) scrambled hypoxic siRNA control: *, p < 0.05; **, p < 0.01; ***, p < 0.001; NS, not significant.

ES and OS cell lines responded in an opposing manner with respect to cell migration during exposure to 18 h hypoxia. ES cell migration was inhibited by 65-80% under hypoxia, whereas OS cells showed a trend to increased hypoxic migration (Figure 6A). Despite contrasting responses to the initial stimulus, both ES and OS cells demonstrated similar responses to HIF siRNA, with targeted inhibition of HIF-2α resulting in increased cell migration under hypoxia (Figure 6B).

## Discussion

These results provide the first detailed description of HIF induction by micro-environmental conditions relevant to primary bone tumours in Ewing's sarcoma and osteosarcoma. We have described phenotypic effects of these conditions, outlining the HIF dependence of these phenotypes and differences between the two tumour types regarding these responses.

Work in many cell types describes HIF-1α as constitutively hypoxia-regulated and HIF-2α as more cell type specific, its transcriptional activation being influenced by micro-environmental conditions other than hypoxia [30,31]. Despite both isoforms sharing an identical core binding motif, the majority of HIF-regulated hypoxia-inducible genes are induced by HIF-1α alone or by a combination of both HIF-1α and HIF-2α [27,31-33]. Our results in ES cells reflect this, with HIF-regulated expression of VEGF, Glut-1 and PGK (the luciferase assay HRE) being mediated solely by HIF-1α. Also in accordance with previous results [32,34], inducible HIF-2α stimulated expression of HIF-2-specific genes, e.g. TGFα, suggesting that HIF-2α is functional in ES cells but that its effects are normally masked by HIF-1α to regulate the majority of HIF-dependent genes. In contrast, gene expression in OS cells was regulated by both HIF isoforms. Indeed, the observed shift towards relative transcriptional domination of HIF-2α places OS cells in a small group, including 786-0 renal cancer cells and the MDA-MB-435 melanoma cell line [27,28], displaying preferential utilisation of HIF-2α.

Hypoglycaemic induction of HIF-2α is the second transcriptional feature distinguishing ES and OS cell lines. Reports of HIF pathway induction under low glucose conditions suggest a specific role for HIF-2α, for example in neuroblastoma cell lines [35]. Similarly, embryonic stem cells and pancreatic cancer cells under hypoglycaemia induced HIF target gene expression in a manner dependent on the HIFα dimerisation partner ARNT [36], but independent of HIF-1α [37]. Our results in ES cell lines also suggest a dominant role for HIF-2α in regulation of the HIF response to hypoglycaemia. Hypoxic stabilisation of HIF-1α is dependent on the presence of glucose [38], which regulates translation via effects on the mTOR/Akt pathway [39,40]. The lack of a HIF transcriptional response to low glucose in OS cells may represent a differential sensitivity to glucose deprivation. This could be a consequence of the high levels of PAS^+ ^glycogen present in ES cells [41] that might provide sufficient intracellular available glucose for translation of HIF under hypoglycaemic conditions.

Phenotypic effects of prolonged exposure to hypoxia and low glucose were similar in both cell types. Hypoxia did not affect cell number, despite evidence of increased apoptosis. Hypoglycaemia produced a ≥ 45% reduction in cell number, predominantly due to reduced cellular proliferation. The only previous reports on apoptosis in ES cell lines described A673 as resistant [23] and SK-N-MC as sensitive [42] to hypoxia-induced apoptosis. Our results for A673 and SK-N-MC show a 2 to 3-fold increase in apoptosis after 72 h at 0.1% O_2_, longer exposure to more severe hypoxia potentially explaining the increased apoptosis in A673. Hypoxia-induced apoptosis of OS cells has not previously been reported, but was generally 2-fold greater than that in ES cell lines. There is considerably less data regarding hypoglycaemia-induced apoptosis [43], suggesting that this is potentially a cell type-specific response which was largely absent from either ES or OS cell lines.

It is intriguing that both ES and OS cells were sensitive to hypoxia-induced apoptosis but that HIF siRNA had no effect on the apoptotic phenotype. In the majority of cells, hypoxia-induced apoptosis is mediated by HIF-1α [23,44,45]. However there is an increasing literature suggesting that, in some cell types, apoptosis induced by severe hypoxia is HIF-1α-independent [46,47]. However, in ES *in vivo *expression of HIF-1α and cleaved caspase 3 localised to areas adjacent to necrotic, and presumably hypoxic, tissue and HIF-1α has been correlated with the percentage of dead cells in OS tumours [6]., This suggests some *in vivo *relationship between HIF and apoptosis which we have been unable to dissect in the *in vitro *situation.

The first phenotypic difference between ES and OS cell lines was the effect of HIF siRNA on hypoxic proliferation. Although overall proliferation rates were similar in normoxia and hypoxia in both cell types, HIF-1α siRNA enhanced proliferation 2 to 3-fold in ES cells whereas in 2/3 OS cell lines both HIF-1α and HIF-2α siRNA inhibited hypoxic proliferation. It is not uncommon for HIF-1α and HIF-2α to exert opposing phenotypic effects in different cell lines. For example, HIF-2α is necessary to maintain tumour growth in RCC4 renal carcinoma cells [48], potentially via regulation of the cell cycle regulatory proteins TGFα and cyclin D1. It may also specifically promote proliferation by enhancing activity of the c-Myc oncoprotein [49], in contrast to inhibition of cell cycle progression by HIF-1α counteracting c-Myc transcriptional activity [50]. Conversely, in SW480 colon cancer cells, HIF-1α siRNA inhibits proliferation whereas HIF-2α siRNA increases anchorage independent growth [51].

The second phenotypic difference between the two cell types encompassed hypoxic migration, which was inhibited in ES cells and maintained or increased in OS cell lines. In both cell types, however, HIF-2α siRNA stimulated hypoxic migration, suggesting an effect of HIF-2α to inhibit cell migration under hypoxia. There are many reports demonstrating migration to be dependent on either HIF-1α and/or HIF-2α in different cell types [27,51,52], again demonstrating the ability of HIF-1α and HIF-2α to exert distinct phenotypic effects in different cell lines.

This data implicates hypoxia as the primary micro-environmental stimulus inducing expression of HIF and downstream genes in both ES and OS cells in culture. However, phenotypic results in both cell types suggest that hypoxia does not significantly increase either cell number or migration and, in addition, enhances tumour cell apoptosis. This does not correlate with clinical reports that hypoxia [9,16-18] and/or HIF-1α [6-8] are predictive of poor outcome in these tumours. This may be due to antagonistic effects of genes downstream of HIF, which cancel each other out when manipulating the whole pathway *in vitro*. For example, VEGF blockade decelerates the growth of experimental osteosarcoma [53] and Ewing's sarcoma [54], demonstrating the viablility of specific targeting of HIF downstream genes. Despite being currently unable to dissect specific mechanisms by which HIF promotes ES and OS progression *in vivo*, we have identified features of the HIF pathway which distinguish the two cell types. This provides a solid platform from which to advance our understanding of the role of hypoxia and HIF in the biology of primary bone sarcomas.

## Conclusions

To our knowledge, this study represents the first comprehensive report of effects of hypoxia and hypoglycaemia on HIF expression, transcriptional activation and phenotype of ES and OS cells. The two cell types differed with respect to their HIF isoform-specific transcriptional response to hypoxia and hypoglycaemia. Phenotypically, ES and OS cells demonstrated opposing migratory responses to hypoxia and opposing HIF-specific effects on hypoxic proliferation. Although the specific mechanism(s) whereby HIF promotes ES and OS progression *in vivo *remains to be identified, this data provides a comprehensive characterisation from which to advance our understanding of HIF function in the pathobiology of primary bone sarcomas.

## Competing interests

The authors declare that they have no competing interests.

## Authors' contributions

HJK designed the study, performed the experimental work and wrote the manuscript. KLS constructed the ES tissue microarray. UD collected samples and patient information for the tissue microarray. NAA conceived the study, contributed to the study design and writing of the manuscript and provided additional ES tissue sections. All authors read and approved the final manuscript.

## Pre-publication history

The pre-publication history for this paper can be accessed here:

http://www.biomedcentral.com/1471-2407/10/372/prepub
